# Pre-injury Comorbidities Are Associated With Functional Impairment and Post-concussive Symptoms at 3- and 6-Months After Mild Traumatic Brain Injury: A TRACK-TBI Study

**DOI:** 10.3389/fneur.2019.00343

**Published:** 2019-04-09

**Authors:** John K. Yue, Maryse C. Cnossen, Ethan A. Winkler, Hansen Deng, Ryan R. L. Phelps, Nathan A. Coss, Sourabh Sharma, Caitlin K. Robinson, Catherine G. Suen, Mary J. Vassar, David M. Schnyer, Ava M. Puccio, Raquel C. Gardner, Esther L. Yuh, Pratik Mukherjee, Alex B. Valadka, David O. Okonkwo, Hester F. Lingsma, Geoffrey T. Manley, TRACK-TBI Investigators, Shelly R. Cooper

**Affiliations:** Author Affiliations: Department of Psychology, Washington University in St. Louis, St. Louis, MO, USA; Department of Rehabilitation Medicine, Icahn School of Medicine at Mount Sinai, New York, NY, USA; Department of Rehabilitation Medicine, Icahn School of Medicine at Mount Sinai, New York, NY, USA; Department of Neurosurgery, University of Pittsburgh Medical Center, Pittsburgh, PA, USA; Department of Neurosurgery, University Hospital Antwerp, Edegem, Belgium; Division of Anaesthesia, University of Cambridge, Cambridge, United Kingdom; Department of Neurosurgery, University of California San Francisco, San Francisco, CA, USA.; ^1^Department of Neurosurgery, University of California, San Francisco, San Francisco, CA, United States; ^2^Brain and Spinal Injury Center, Zuckerberg San Francisco General Hospital, San Francisco, CA, United States; ^3^Department of Public Health, Erasmus Medical Center, Rotterdam, Netherlands; ^4^Department of Neurology, University of Utah, Salt Lake City, UT, United States; ^5^Department of Psychology, University of Texas in Austin, Austin, TX, United States; ^6^Department of Neurosurgery, University of Pittsburgh Medical Center, Pittsburgh, PA, United States; ^7^Department of Neurology, University of California, San Francisco, San Francisco, CA, United States; ^8^Department of Neurology, Veterans Affairs Medical Center, San Francisco, CA, United States; ^9^Department of Radiology, University of California, San Francisco, San Francisco, CA, United States; ^10^Department of Neurosurgery, Virginia Commonwealth University, Richmond, VA, United States

**Keywords:** functional impairment, mild traumatic brain injury, post-concussive symptoms, pre-injury comorbidities, prognosis

## Abstract

**Introduction:** Over 70% of traumatic brain injuries (TBI) are classified as mild (mTBI), which present heterogeneously. Associations between pre-injury comorbidities and outcomes are not well-understood, and understanding their status as risk factors may improve mTBI management and prognostication.

**Methods:** mTBI subjects (GCS 13–15) from TRACK-TBI Pilot completing 3- and 6-month functional [Glasgow Outcome Scale-Extended (GOSE)] and post-concussive outcomes [Acute Concussion Evaluation (ACE) physical/cognitive/sleep/emotional subdomains] were extracted. Pre-injury comorbidities >10% incidence were included in regressions for functional disability (GOSE ≤ 6) and post-concussive symptoms by subdomain. Odds ratios (OR) and mean differences (B) were reported. Significance was assessed at *p* < 0.0083 (Bonferroni correction).

**Results:** In 260 subjects sustaining blunt mTBI, mean age was 44.0-years and 70.4% were male. Baseline comorbidities >10% incidence included psychiatric-30.0%, cardiac (hypertension)-23.8%, cardiac (structural/valvular/ischemic)-20.4%, gastrointestinal-15.8%, pulmonary-15.0%, and headache/migraine-11.5%. At 3- and 6-months separately, 30.8% had GOSE ≤ 6. At 3-months, psychiatric (GOSE ≤ 6: OR = 2.75, 95% CI [1.44–5.27]; ACE-physical: B = 1.06 [0.38–1.73]; ACE-cognitive: B = 0.72 [0.26–1.17]; ACE-sleep: B = 0.46 [0.17–0.75]; ACE-emotional: B = 0.64 [0.25–1.03]), headache/migraine (GOSE ≤ 6: OR = 4.10 [1.67–10.07]; ACE-sleep: B = 0.57 [0.15–1.00]; ACE-emotional: B = 0.92 [0.35–1.49]), and gastrointestinal history (ACE-physical: B = 1.25 [0.41–2.10]) were multivariable predictors of worse outcomes. At 6-months, psychiatric (GOSE ≤ 6: OR = 2.57 [1.38–4.77]; ACE-physical: B = 1.38 [0.68–2.09]; ACE-cognitive: B = 0.74 [0.28–1.20]; ACE-sleep: B = 0.51 [0.20–0.83]; ACE-emotional: B = 0.93 [0.53–1.33]), and headache/migraine history (ACE-physical: B = 1.81 [0.79–2.84]) predicted worse outcomes.

**Conclusions:** Pre-injury psychiatric and pre-injury headache/migraine symptoms are risk factors for worse functional and post-concussive outcomes at 3- and 6-months post-mTBI. mTBI patients presenting to acute care should be evaluated for psychiatric and headache/migraine history, with lower thresholds for providing TBI education/resources, surveillance, and follow-up/referrals.

**Clinical Trial Registration:** www.ClinicalTrials.gov, identifier NCT01565551.

## Introduction

Traumatic brain injury (TBI) remains a significant cause of morbidity and mortality worldwide. In 2013 ~2.8 million TBI cases were recorded annually in the United States (U.S.) (1), which is a 160% increase from 2007 (2). Updated estimates suggest closer to 4 million, due to subpopulations of patients who do not seek care due to either inadequate access or perceived lack of need (2–4). Over 70% of TBI is classified as “mild (mTBI)” defined by Glasgow Coma Scale (GCS) score 13-15 (3, 5), which present heterogeneously with a range of demographic and clinical risk factors. Although a substantial portion of mTBI patients fully recover without intervention, up to 50% suffer long-term functional and/or neuropsychological sequelae, leading to a substantial burden on both patients and the healthcare system (3, 6). This heterogeneity poses a problem in the clinic, as some risk factors are conserved while others differ across different outcome instruments. Whether predictors differ across different outcome time points is also unclear, and hence it remains challenging to risk-stratify patients who will benefit most from additional resources and follow-up in both acute and chronic settings after mTBI (7).

In the orthopedic and geriatric literature, it is recognized that pre-existing conditions impact outcomes after acute illness or injury. However,there is a paucity of research investigating the relationship between pre-injury comorbidities and outcome after mTBI. A number of studies have focused exclusively on psychiatric comorbidities, and PTSD, on mTBI outcome (8–12). Some studies consider the number of rather than types of comorbidities (13), while others focus on mTBI as a risk factor for worsened systemic conditions but not the reverse (14–18). Pre-injury comorbidities are routinely collected during standard clinical interview and documented in the medical record, which underscores their utility as a readily available data source in both acute and ambulatory care settings without increasing time or cost burden. Elucidation of the associations between certain pre-injury conditions and domains of outcome will help clinicians and researchers better understand contributors and modifiers of injury in this heterogeneous group of patients, and may improve early risk stratification of and resource allocation for those at risk for unfavorable recovery.

Systemic medical conditions intrinsically influence physical and cognitive reserve at baseline, and may exert differential effects on recovery in the brain-injured patient. To date, many mTBI studies have understandably excluded patients with pre-injury comorbidities to reduce outcome variability when isolating risk factors (19). Unfortunately, this hinders the clinician's approach to complex patients with pre-injury conditions who suffer mTBI. In the current analysis, we characterize the baseline systemic comorbidities of a prospectively collected multicenter mTBI sample with a high prevalence of pre-injury comorbidities, and investigate the relationships between systemic comorbidities and 3- and 6-month functional and post-concussive outcomes.

## Methods

The prospective Transforming Research and Clinical Knowledge in Traumatic Brain Injury Pilot (TRACK-TBI Pilot) study was conducted at three U.S. Level I trauma centers [Zuckerberg San Francisco General Hospital (California), University of Pittsburgh Medical Center (Pennsylvania), University Medical Center Brackenridge (Austin, Texas)] using the National Institute of Neurological Disorders and Stroke (NINDS) TBI Common Data Elements (CDEs) (20–24). Inclusion criteria for TRACK-TBI Pilot were age ≥16-years, external force head trauma, presentation to enrolling center, and clinically-indicated head computed tomography (CT) scan <24 h of injury. Exclusion criteria were pregnancy, ongoing life-threatening disease (e.g., end-stage malignancy), police custody, involuntary psychiatric hold, and non-English speakers due to multiple outcome measures administered and/or normed only in English. As the goal of this analysis was to evaluate the associations between baseline comorbidities and outcomes, subjects with emergency department (ED) admission GCS 13–15 who completed the Glasgow Outcome Scale-Extended (GOSE) and Acute Concussion Evaluation (ACE) at 3- and 6-months were included. To minimize confounding of TBI outcomes, subjects with history of central nervous system malignancy, cerebrovascular anomaly/accident, human immunodeficiency virus/acquired immunodeficiency syndrome, and/or developmental delay were excluded.

Eligible subjects were enrolled by convenience sampling from years 2010–2012. Institutional Review Board approval was obtained at each participating site. Informed consent was obtained prior to enrollment. For subjects unable to provide consent due to injury, surrogate consent was obtained. Subjects were re-consented, if cognitively able, during the course of clinical care and/or follow-up timepoints for study participation.

### Demographic and Clinical Variables

Subjects underwent a baseline assessment at ED admission. Variables were collected according to NINDS CDE version 1 (21, 23, 24). Twelve CDE pre-injury comorbidity categories (i.e., comorbidities present at baseline prior to the index mTBI of enrollment) were collected by standard checklists through self-report and chart abstraction, including cardiac-hypertension, cardiac-structural/ischemic/valvular, diabetes mellitus, gastrointestinal, hematologic, headache/migraine, hepatic, pulmonary, psychiatric, renal, seizure, and thyroid.

### Outcome Measures

Outcome measures were collected through in-person or phone interview at 3- and 6-months. To focus on functional disability and post-concussive symptoms, the following measures were analyzed:

Glasgow Outcome Scale-Extended (GOSE): Structured interview which provides an overall measure of disability based on cognition, independence, employability, and social/community participation, and has been widely used as a standard outcome measure for TBI studies (25). Scores include: 1 = dead, 2 = vegetative state, 3 = lower severe disability, 4 = upper severe disability, 5 = lower moderate disability, 6 = upper moderate disability, 7 = lower good recovery, and 8 = upper good recovery. A score of 8 reflects recovery to baseline without new disability. For the current analysis, the ordinal GOSE was dichotomized into “good recovery (GOSE 7–8)” vs. “moderate disability or worse (GOSE ≤ 6),” consistent with prior reports (26, 27).

Acute Concussion Evaluation (ACE): First reported by a consensus sports neuropsychology panel in 1998 and adopted by the U.S. Centers for Disease Control and Prevention (CDC) in 2006 (28, 29). It contains 22 specific post-concussive symptoms classified into 4 domains: physical (10 symptoms), cognitive (4 symptoms), sleep (4 symptoms), and emotional (4 symptoms). Subjects were queried regarding the presence/absence of each symptom and the number of symptoms per domain were totaled for analysis.

### Statistical Analysis

Descriptive statistics were assessed using means and standard deviations (SD) for continuous variables and proportions for categorical variables. Three- and six-month functional outcomes were analyzed using logistic regression (GOSE ≤ 6 vs. 7–8), and post-concussive outcomes were analyzed by domain using linear regression (number of symptoms). As variables of interest, pre-injury comorbidities with >10% incidence were included in multivariable models for outcome, controlling for age, sex, education (years), ED admission GCS, and presence/absence of intracranial abnormalities on CT. Multivariable odds ratios (OR) and associated 95% confidence intervals (CI) were reported for each predictor. Significance was assessed at *p* < 0.0083 using the Bonferroni correction (0.05 ÷ 6 comorbidities). Analyses were performed using Statistical Package for the Social Sciences (SPSS) version 24 (IBM Corp., Chicago, IL).

## Results

Overall, 260 mTBI subjects had a mean age of 44.0 ± 18.7-years, 70.4% were male, 78.6% were Caucasian, and 42.3% were head CT+. Baseline comorbidities >10% incidence included psychiatric (30.0%), cardiac-hypertension (23.8%), cardiac-structural/valvular/ischemic (20.4%), gastrointestinal (15.8%), pulmonary (15.0%), and headache/migraine (11.5%) (Table 1). At 3- and 6-months, 30.8% had GOSE ≤ 6. Additional demographic and clinical variables are shown in Table 1.

**Table 1:** Demographic and clinical characteristics in 260 mTBI subjects.

**Variable**	***N* (%) or mean ± SD**
**AGE**	
Years (mean, SD)	44.0 ± 18.7
**SEX**	
Male	183 (70.4%)
Female	77 (29.6%)
**EDUCATION**	
Years (mean, SD)	14.2 ± 2.9
**MECHANISM OF INJURY**	
Motor vehicle accident	61 (23.5%)
Pedestrian vs. auto	37 (14.2%)
Fall	117 (45.0%)
Assault	35 (13.5%)
Struck by object	10 (3.9%)
**ED ADMISSION GCS**	
13	9 (3.5%)
14	53 (20.4%)
15	198 (70.2%)
**INTRACRANIAL CT FINDINGS**	
No	150 (57.7%)
Yes	110 (42.3%)
**ED DISPOSITION**	
Discharge home	88 (38.8%)
Hospital ward admit	109 (41.9%)
ICU admit	63 (24.2%)
**PRE-INJURY COMORBIDITIES**	
Psychiatric history	78 (30.0%)
Cardiac-hypertension history	62 (23.8%)
Cardiac-structural/ischemic/valvular history	53 (20.4%)
Gastrointestinal history	41 (15.8%)
Pulmonary history	39 (15.0%)
Headache/migraine history	30 (11.5%)
Seizure history	22 (8.5%)
Diabetes history	21 (8.1%)
Hepatic history	20 (7.7%)
Renal history	15 (5.8%)
Thyroid history	13 (5.0%)
Hematologic history	12 (4.6%)
**3-MONTHS OUTCOMES**	
GOSE ≤ 6	80 (30.8%)
ACE physical	2.33 ± 2.56
ACE cognitive	0.94 ± 1.10
ACE sleep	1.38 ± 1.65
ACE emotional	1.05 ± 1.45
**6-MONTHS OUTCOMES**	
GOSE ≤ 6	80 (30.8%)
ACE physical	2.84 ± 2.74
ACE cognitive	1.22 ± 1.18
ACE sleep	1.70 ± 1.69
ACE emotional	1.42 ± 1.52

*ACE, Acute Concussion Evaluation; CT, computed tomography; ED, emergency department; GCS, Glasgow Coma Scale; GOSE, Glasgow Outcome Scale Extended; ICU, intensive care unit; SD, standard deviation*.

On multivariable analysis at 3-months, psychiatric history was a predictor for functional disability (GOSE ≤ 6: OR = 2.75, 95% CI [1.44–5.27]) and all domains of post-concussive symptoms (ACE-physical: B = 1.06 [0.38–1.73]; ACE-cognitive: B = 0.72 [0.26–1.17]; ACE-sleep: B = 0.46 [0.17–0.75]; ACE-emotional: B = 0.64 [0.25–1.03]). Headaches/migraine history was a predictor for functional disability (GOSE ≤ 6: OR = 4.10 [1.67–10.07]), and sleep and emotional post-concussive symptoms (ACE-sleep: B = 0.57 [0.15–1.00]; ACE-emotional: B = 0.92 [0.35–1.49]). Gastrointestinal history was a predictor for physical post-concussive symptoms (ACE-physical: B = 1.25 [0.41–2.10]) (Table 2A).

**Table 2A:** Multivariable regression of 3-months outcomes.

**Variable**	**GOSE ≤ 6 (3-months)**		**ACE physical (3-months)**		**ACE cognitive (3-months)**		**ACE sleep (3-months)**		**ACE emotional (3-months)**	
	**OR [95% CI]**	**Sig. (*p*)**	**OR [95% CI]**	**Sig. (*p*)**	**OR [95% CI]**	**Sig. (*p*)**	**OR [95% CI]**	**Sig. (*p*)**	**OR [95% CI]**	**Sig. (*p*)**
**AGE**										
Per-year	1.017 [0.997, 1.037]	0.089	0.007 [−0.013, 0.027]	0.478	0.006 [−0.008, 0.019]	0.398	0.001 [−0.008, 0.009]	0.854	0.003 [−0.009, 0.014]	0.629
**SEX**										
Male	Reference	–	Reference	–	Reference	–	Reference	–	Reference	–
Female	0.94 [0.48, 1.83]	0.855	0.53 [−0.15, 1.20]	0.124	−0.01 [−0.46, 0.44]	0.955	0.10 [−0.20, 0.39]	0.518	0.25 [−0.14, 0.65]	0.201
**EDUCATION**										
Per–year	0.98 [0.88, 1.09]	0.698	−0.15 [−0.26, −0.05]	0.005	−0.03 [−0.10, 0.04]	0.429	−0.06 [−0.10, −0.01]	0.015	−0.06 [−0.12, 0.00]	0.059
**ED GCS**										
= 15	Reference	–	Reference	–	Reference	–	Reference	–	Reference	–
= 13–14	1.17 [0.59, 2.33]	0.653	0.52 [−0.18, 1.22]	0.147	0.33 [−0.14, 0.80]	0.171	0.21 [−0.09, 0.51]	0.175	0.13 [−0.28, 0.53]	0.537
**CT INTRACRANIAL LESION**										
Absent	Reference	–	Reference	–	Reference	–	Reference	–	Reference	–
Present	3.12 [1.62, 6.02]	0.001	0.35 [−0.30, 1.01]	0.289	0.36 [−0.08, 0.80]	0.104	0.23 [−0.05, 0.52]	0.107	0.09 [−0.29, 0.47]	0.653
**CARDIAC-HYPERTENSION HISTORY**										
Absent	Reference	–	Reference	–	Reference	–	Reference	–	Reference	–
Present	0.55 [0.23, 1.29]	0.168	0.29 [−0.54, 1.12]	0.493	−0.07 [−0.63, 0.49]	0.813	−0.01 [−0.37, 0.35]	0.949	0.20 [−0.28, 0.68]	0.412
**CARDIAC-STRUCTURAL/ISCHEMIC/VALVULAR HISTORY**										
Absent	Reference	–	Reference	–	Reference	–	Reference	–	Reference	–
Present	0.68 [0.30, 1.51]	0.338	−0.98 [−1.84, 0.12]	0.026	−0.12 [−0.70, 0.46]	0.685	−0.19 [−0.56, 0.18]	0.317	−0.32 [−0.82, 0.18]	0.206
**GASTROINTESTINAL HISTORY**										
Absent	Reference	–	Reference	–	Reference	–	Reference	–	Reference	–
Present	1.56 [0.71, 3.43]	0.265	1.25 [0.41, 2.10]	0.004	0.36 [−0.21, 0.92]	0.218	0.44 [0.07, 0.80]	0.019	0.23 [−0.26, 0.72]	0.358
**PULMONARY HISTORY**										
Absent	Reference	–	Reference	–	Reference	–	Reference	–	Reference	–
Present	0.40 [0.15, 1.07]	0.068	−0.68 [−1.55, 0.20]	0.129	−0.48 [−1.06, 0.11]	0.109	−0.44 [−0.82, −0.06]	0.023	−0.21 [−0.72, 0.30]	0.412
**PSYCHIATRIC HISTORY**										
Absent	Reference	–	Reference	–	Reference	–	Reference	–	Reference	–
Present	2.75 [1.44, 5.27]	0.002	1.06 [0.38, 1.73]	0.002	0.72 [0.26, 1.17]	0.002	0.46 [0.17, 0.75]	0.002	0.64 [0.25, 1.03]	0.002
**HEADACHE/MIGRAINE HISTORY**										
Absent	Reference	–	Reference	–	Reference	–	Reference	–	Reference	–
Present	4.10 [1.67, 10.07]	0.002	0.86 [−0.12, 1.84]	0.085	0.76 [0.10, 1.42]	0.023	0.57 [0.15, 1.00]	0.008	0.92 [0.35, 1.49]	0.002

*Significance set at p < 0.0083. ACE, Acute Concussion Evaluation; CI, confidence interval; CT, computed tomography; ED, emergency department; GCS, Glasgow Coma Scale; GOSE, Glasgow Outcome Scale Extended; OR, odds ratio*.

On multivariable analysis at 6-months, psychiatric history was a predictor for functional disability (GOSE ≤ 6: OR = 2.57 [1.38–4.77]) and all domains of post-concussive symptoms (ACE-physical: B = 1.38 [0.68–2.09]; ACE-cognition: B = 0.74 [0.28–1.20]; ACE-sleep: B = 0.51 [0.20–0.83]; ACE-emotional: B = 0.93 [0.53–1.33]). Headache/migraine history was a predictor for physical post-concussive symptoms (ACE-physical: B = 1.81 [0.79–2.84]) (Table 2B).

**Table 2B:** Multivariable regression of 6-months outcomes.

**Variable**	**GOSE≤6 (6-months)**		**ACE physical (6-months)**		**ACE cognitive (6-months)**		**ACE sleep (6-months)**		**ACE emotional (6-months)**	
	**OR [95% CI]**	**Sig. (*p*)**	**OR [95% CI]**	**Sig. (*p*)**	**OR [95% CI]**	**Sig. (*p*)**	**OR [95% CI]**	**Sig. (*p*)**	**OR [95% CI]**	**Sig. (*p*)**
**AGE**										
Per-year	1.007 [0.988, 1.026]	0.487	0.016 [−0.005, 0.037]	0.127	−0.003 [−0.016, 0.011]	0.670	0.010 [0.000, 0.019]	0.041	−0.007 [−0.019, 0.005]	0.254
**SEX**										
Male	Reference	–	Reference	–	Reference	–	Reference	–	Reference	–
Female	1.44 [0.76, 2.74]	0.259	0.89 [0.19, 1.60]	0.013	0.08 [−0.38, 0.53]	0.744	0.03 [−0.28, 0.35]	0.838	0.22 [−0.19, 0.62]	0.290
**EDUCATION**										
Per-year	0.90 [0.81, 0.99]	0.036	−0.08 [−0.19, 0.03]	0.147	−0.04 [−0.11, 0.04]	0.313	−0.05 [−0.10, 0.00]	0.035	−0.02 [−0.09, 0.04]	0.475
**ED GCS**										
= 15	Reference	–	Reference	–	Reference	–	Reference	–	Reference	–
= 13–14	2.27 [1.20, 4.35]	0.012	0.73 [−0.01, 1.46]	0.053	0.49 [0.01, 0.96]	0.044	0.32 [−0.01, 0.65]	0.057	0.47 [0.05, 0.89]	0.029
**CT INTRACRANIAL LESION**										
Absent	Reference	–	Reference	–	Reference	–	Reference	–	Reference	–
Present	1.44 [0.77, 2.70]	0.260	−0.38 [−1.07, 0.30]	0.273	0.32 [−0.12, 0.76]	0.156	−0.37 [−0.67, −0.06]	0.018	−0.17 [−0.57, 0.22]	0.386
**CARDIAC–HYPERTENSION HISTORY**										
Absent	Reference	–	Reference	–	Reference	–	Reference	–	Reference	–
Present	0.53 [0.22, 1.25]	0.145	0.13 [−0.74, 1.01]	0.762	0.01 [−0.55, 0.57]	0.971	−0.15, [−0.53, 0.24]	0.462	0.00 [−0.50, 0.50]	0.987
**CARDIAC-STRUCTURAL/ISCHEMIC/VALVULAR HISTORY**										
Absent	Reference	–	Reference	–	Reference	–	Reference	–	Reference	–
Present	0.99 [0.45, 2.15]	0.972	−0.39 [−1.29, 0.51]	0.396	0.12 [−0.46, 0.71]	0.674	−0.16 [−0.56, 0.24]	0.428	0.22 [−0.29, 0.74]	0.396
**GASTROINTESTINAL HISTORY**										
Absent	Reference	–	Reference	–	Reference	–	Reference	–	Reference	–
Present	1.89 [0.87, 4.11]	0.107	0.61 [−0.27, 1.50]	0.173	0.72 [0.15, 1.29]	0.014	0.37 [−0.02, 0.76]	0.065	0.42 [−0.09, 0.92]	0.106
**PULMONARY HISTORY**										
Absent	Reference	–	Reference	–	Reference	–	Reference	–	Reference	–
Present	0.87 [0.37, 2.03]	0.740	−0.46 [−1.37, 0.45]	0.323	−0.10 [−0.69, 0.49]	0.743	0.18 [−0.22, 0.59]	0.365	−0.54 [−1.06, −0.02]	0.043
**PSYCHIATRIC HISTORY**										
Absent	Reference	–	Reference	–	Reference	–	Reference	–	Reference	–
Present	2.57 [1.38, 4.77]	0.003	1.38 [0.68, 2.09]	< 0.001	0.74 [0.28, 1.20]	0.001	0.51 [0.20, 0.83]	0.001	0.93 [0.53, 1.33]	< 0.001
**HEADACHE/MIGRAINE HISTORY**										
Absent	Reference	–	Reference	–	Reference	–	Reference	–	Reference	–
Present	1.72 [0.71, 4.13]	0.227	1.81 [0.79, 2.84]	0.001	0.78 [0.12, 1.44]	0.021	0.26 [−0.19, 0.72]	0.259	0.43 [0.16, 1.02]	0.150

*Significance set at p < 0.0083. ACE, Acute Concussion Evaluation; CI, confidence interval; CT, computed tomography; ED, emergency department; GCS, Glasgow Coma Scale; GOSE, Glasgow Outcome Scale Extended; OR, odds ratio*.

## Discussion

The heterogeneity of mTBI in risk factors and outcomes leads to clinical challenges in patient-specific triage, treatment and prognosis. In a comprehensive report of pre-injury comorbidities and mTBI, we found psychiatric, cardiac, gastrointestinal, pulmonary, and headache/migraine comorbidities to be of the highest incidence. In 260 mTBI subjects, psychiatric history was a predictor of functional disability and increased post-concussive at 3- and 6-months controlling for demographic and clinical variables and other pre-injury comorbidities. Additionally, headache/migraine history was a predictor of functional disability, sleep and emotional symptoms at 3-months, and physical symptoms at 6-months. These results constitute a first step to improved understanding of pre-injury risk factors and improved awareness of subsets of patients who may benefit from careful history taking, increased education, and surveillance, including triage to follow-up at early time points.

Psychiatric history is a known predictor of worsened functional and post-concussive outcome after mTBI (30). Over the past decade, studies have shown that baseline psychiatric morbidity is predictive of 2-week and 6-month outcomes separately. The multicenter UPFRONT study in the Netherlands showed that baseline mental health disorders conferred OR 0.31–0.39 for complete functional recovery (GOSE = 8) at 6-months (31). Our study shows that not only is functional recovery more likely to be incomplete in those with psychiatric history, but the effect of OR 2.5–2.8 for moderate functional disability or worse, e.g., unable to return to work, significant social or emotional disruption, is conserved at 3- and 6-months, in addition to the 0.5 to 1.4 more symptoms across post-concussive symptoms domains. Recent studies illustrate that recovery from mTBI is a non-linear process with subgroups of patients failing to rebound from their injury, such that prognostic models using pre-injury risk factors can be constructed to guide post-injury management (32–34). As mTBI patients are increasingly shown to have impairments in cognitive and neuropsychiatric recovery, it becomes ever more important to document and have an accurate understanding of the patient's baseline cognitive, psychiatric, and mental health in order to both monitor post-injury return to baseline, and address deficits from baseline during the process of recovery. Pertinent first steps include documentation of priority pre-injury comorbidities including presence and frequency of *prior* psychiatric and headache symptoms, setting expectations by informing patients with these comorbidities that their symptoms often worsen after mTBI, providing discharge instructions for patients and physicians to monitor whether post-injury symptomatology are new or worse during acute follow-up, and having a lower threshold to refer patients to follow-up with primary or specialist care.

Headache is the most common post-concussive symptom manifestation with 30–90% incidence (35–38). While post-traumatic headache (PTH) is well-documented after mTBI (39, 40), the relationship between pre-injury headaches/migraines with long-term post-injury functional outcomes remains understudied. Improved understanding of this association will help to determine whether a patient's PTH should be considered a new entity vs. a possible exacerbation of baseline headaches after mTBI. We showed that at baseline, 11.5% of patients suffered from headache/migraine, which predicted post-injury functional disability and sleep and emotional post-concussive symptoms at 3-months, as well as physical post-concussive symptoms at 6-months. Our results not only support previous findings regarding the importance of evaluating for premorbid headache/migraine as a risk factor for PTH (40), but also show that headache/migraine history is associated with multiple outcome domains after mTBI. In addition to psychological factors and mental health as predictors of 6-months outcome after mTBI (31), we demonstrate the need to identify risk factors from other categories of pre-injury medical history. In our study, pre-injury headache/migraine was associated with different outcome domains between 3- and 6-months, suggesting that deficits may continue to evolve over time after mTBI. These findings alert both clinicians and researchers to the need for standardized assessment of functional disability and post-concussive symptoms at multiple (and earlier) time points, as early interventions post-injury may decrease maladaptive coping methods, loss of livelihood/productivity, and healthcare costs. Along with psychiatric history and pre-injury headache/migraine being predictors of functional disability and post-concussive symptoms at 3-months, we found that gastrointestinal history also associated with physical post-concussive symptoms. Aside from cognitive and neuropsychiatric impairments, there is emerging interest in understanding systemic effects of autonomic dysfunction after mTBI (41). For the first 3-months post-injury, mTBI can possibly exacerbate the complex and non-specific nature of gastrointestinal symptoms as reflected through physical post-concussive symptoms in the patient.

### Limitations

We studied associations between pre-injury comorbidities and outcomes, and in consideration for not overfitting our regression models, we limited to controlling for known predictors of mTBI outcomes rather than all possible predictors available in our dataset. We did not study trajectories of outcomes, nor whether mTBI had effects on the severity of pre-injury comorbidities. Patient recruitment is limited to Level I trauma centers capturing a more urbanized population, and thus our findings cannot be extrapolated to all mTBI patients. Proportions of telephone vs. in-person follow-ups, which have been shown to influence extent of disclosure in cancer and genetics studies (42), were unavailable from our dataset and constitutes another limitation. This is a study of association, hence we are unable to make claims regarding causality or pathophysiology. We limited our multivariable analysis to comorbidities with over 10% incidence in the sample to provide reliable odds ratios, and future studies of larger sample size will enable analyses of the relationship between specific comorbidities within each organ system and outcome. Lastly, we were limited by the variables available and were unable to investigate whether subjects successfully triaged to and/or completed rehabilitation programs. Our goal is to establish a first step in assessing the importance of baseline comorbidities on mTBI outcome, hence our findings remain exploratory and in need of validation by future trials. Integrating the evaluation of pre-injury comorbidities with that of other baseline predictors not routinely collected on admission, such as education level, may be important in the prognostication of outcome after mTBI.

## Conclusions

Amongst pre-injury comorbidities, history of psychiatric disorder is a risk factor for decreased functional outcome and increased post-concussive symptoms across multiple domains, at 3- and 6-months post-injury after mTBI. History of headache/migraine may also be a risk factor for decreased functional outcome and increased post-concussive symptoms. mTBI patients presenting to acute and post-discharge care should be evaluated for history of baseline psychiatric and headache/migraine disorders, with lower thresholds for provision of TBI education and resources, surveillance, and follow-up/referrals to primary and specialist care.

## Ethics Statement

This study was carried out in accordance with the recommendations of the University of California San Francisco (UCSF) Institutional Review Board of record, the Committee on Human Research (CHR), with written informed consent from all subjects. All subjects gave written informed consent in accordance with the Declaration of Helsinki. The protocol was approved by the UCSF CHR #10-00011.

## Author Contributions

JY, MC, DS, AP, RG, EY, PM, AV, DO, HL, and GM: conception or design of work; JY, MC, EW, HD, RP, NC, SS, CR, CS, MV, DS, AP, RG, EY, PM, AV, DO, HL, and GM: acquisition, analysis, or interpretation of data for the work, providing approval for publication of the content, and agree to be held accountable for all aspects of the work in ensuring that questions related to the accuracy or integrity of any part of the work are appropriately investigated and resolved; JY, MC, EW, HD, RP, NC, SS, CR, CS, MV, DS, AP, EY, RG, PM, AV, DO, HL, and GM: drafting the work or revising it critically for important intellectual content.

### Conflict of Interest Statement

The authors declare that the research was conducted in the absence of any commercial or financial relationships that could be construed as a potential conflict of interest.

## References

[1] Taylor CA Bell JM Breiding MJ Xu L. Traumatic Brain injury-related emergency department visits, hospitalizations, and deaths–United States, 2007 and 2013. MMWR Surveill Summ. (2017) 66:1–16. 10.15585/mmwr.ss6609a1 28301451 PMC5829835

[2] Coronado VG Xu L Basavaraju SV McGuire LC Wald MM Faul MD. Surveillance for traumatic brain injury-related deaths–United States, 1997-2007. MMWR Surveill Summ. (2011) 60:1–32. 21544045

[3] Bruns J Jr, Hauser WA. The epidemiology of traumatic brain injury: a review. Epilepsia. (2003) 44:2–10. 10.1046/j.1528-1157.44.s10.3.x 14511388

[4] Gouvier WD Uddo-Crane M Brown LM. Base rates of post-concussional symptoms. Arch Clin Neuropsychol. (1988) 3:273–8. 10.1093/arclin/3.3.273 14589697

[5] Cassidy JD Carroll LJ Peloso PM Borg J von Holst H Holm L Incidence, risk factors and prevention of mild traumatic brain injury: results of the WHO Collaborating Centre Task Force on Mild Traumatic Brain Injury. J Rehabil Med. (2004) 43(Suppl.):28–60. 10.1080/16501960410023732 15083870

[6] Fleminger S Ponsford J. Long term outcome after traumatic brain injury. BMJ. (2005) 331:1419–20. 10.1136/bmj.331.7530.1419 16356951 PMC1315633

[7] Jagoda AS Bazarian JJ Bruns JJ Jr. Cantrill SV Gean AD Howard PK. Clinical policy: neuroimaging and decisionmaking in adult mild traumatic brain injury in the acute setting. J Emerg Nurs. (2009) 35:e5–40. 10.1016/j.jen.2008.12.010 19285163

[8] Stein MB Ursano RJ Campbell-Sills L Colpe LJ Fullerton CS Heeringa SG. Prognostic indicators of persistent post-concussive symptoms after deployment-related mild traumatic brain injury: a prospective longitudinal study in U.S. Army Soldiers. J Neurotrauma. (2016) 33:2125–32. 10.1089/neu.2015.4320 26905672 PMC5124734

[9] Spielberg JM McGlinchey RE Milberg WP Salat DH. Brain network disturbance related to posttraumatic stress and traumatic brain injury in veterans. Biol Psychiatry. (2015) 78:210–6. 10.1016/j.biopsych.2015.02.013 25818631

[10] Walker WC Franke LM McDonald SD Sima AP Keyser-Marcus L. Prevalence of mental health conditions after military blast exposure, their co-occurrence, and their relation to mild traumatic brain injury. Brain Inj. (2015) 29:1581–8. 10.3109/02699052.2015.1075151 26479126

[11] Amick MM Clark A Fortier CB Esterman M Rasmusson AM Kenna A. PTSD modifies performance on a task of affective executive control among deployed OEF/OIF veterans with mild traumatic brain injury. J Int Neuropsychol Soc. (2013) 19:792–801. 10.1017/S1355617713000544 23823533 PMC4003877

[12] Davenport ND Lamberty GJ Nelson NW Lim KO Armstrong MT Sponheim SR. PTSD confounds detection of compromised cerebral white matter integrity in military veterans reporting a history of mild traumatic brain injury. Brain Inj. (2016) 30:1491–500. 10.1080/02699052.2016.1219057 27834537

[13] Theadom A Parag V Dowell T McPherson K Starkey N Barker-Collo S. Persistent problems 1 year after mild traumatic brain injury: a longitudinal population study in New Zealand. Br J Gen Pract. (2016) 66:e16–23. 10.3399/bjgp16X683161 26719482 PMC4684031

[14] Ahmadi N Hajsadeghi F Yehuda R Anderson N Garfield D Ludmer C. Traumatic brain injury, coronary atherosclerosis and cardiovascular mortality. Brain Inj. (2015) 29:1635–41. 10.3109/02699052.2015.1075149 26399477

[15] Heltemes KJ Dougherty AL MacGregor AJ Galarneau MR. Alcohol abuse disorders among U.S. service members with mild traumatic brain injury. Mil Med. (2011) 176:147–50. 10.7205/MILMED-D-10-00191 21366075

[16] Lucas S. Posttraumatic headache: clinical characterization and management. Curr Pain Headache Rep. (2015) 19:48. 10.1007/s11916-015-0520-1 26280569

[17] Vanderploeg RD Curtiss G Luis CA Salazar AM. Long-term morbidities following self-reported mild traumatic brain injury. J Clin Exp Neuropsychol. (2007) 29:585–98. 10.1080/13803390600826587 17691031

[18] Jodoin M Rouleau DM Gosselin N Benoit B Leduc S Laflamme Y. Comorbid mild traumatic brain injury increases pain symptoms in patients suffering from an isolated limb fracture. Injury. (2017) 48:1927–31. 10.1016/j.injury.2017.06.025 28693815

[19] Isokuortti H Iverson GL Kataja A Brander A Öhman J Luoto TM Who gets head trauma or recruited in mild traumatic brain injury research? J Neurotrauma. (2016) 33:232–41. 10.1089/neu.2015.3888 26054639

[20] Duhaime A-C Gean AD Haacke EM Hicks R Wintermark M Mukherjee P. Common data elements in radiologic imaging of traumatic brain injury. Arch Phys Med Rehabil. (2010) 91:1661–6. 10.1016/j.apmr.2010.07.238 21044709

[21] Maas AI Harrison-Felix CL Menon D Adelson PD Balkin T Bullock R. Common data elements for traumatic brain injury: recommendations from the interagency working group on demographics and clinical assessment. Arch Phys Med Rehabil. (2010) 91:1641–9. 10.1016/j.apmr.2010.07.232 21044707

[22] Manley GT Diaz-Arrastia R Brophy M Engel D Goodman C Gwinn K. Common data elements for traumatic brain injury: recommendations from the biospecimens and biomarkers working group. Arch Phys Med Rehabil. (2010) 91:1667–72. 10.1016/j.apmr.2010.05.018 21044710

[23] Wilde EA Whiteneck GG Bogner J Bushnik T Cifu DX Dikmen S. Recommendations for the use of common outcome measures in traumatic brain injury research. Arch Phys Med Rehabil. (2010) 91:1650–60.e17. 10.1016/j.apmr.2010.06.033 21044708

[24] Yue JK Vassar MJ Lingsma HF Cooper SR Okonkwo DO Valadka AB. Transforming research and clinical knowledge in traumatic brain injury pilot: multicenter implementation of the common data elements for traumatic brain injury. J Neurotrauma. (2013) 30:1831–44. 10.1089/neu.2013.2970 23815563 PMC3814815

[25] Wilson JT Pettigrew LE Teasdale GM. Structured interviews for the Glasgow Outcome Scale and the extended Glasgow Outcome Scale: guidelines for their use. J Neurotrauma. (1998) 15:573–85. 10.1089/neu.1998.15.573 9726257

[26] Jacobs B Beems T Stulemeijer M van Vugt AB van der Vliet TM Borm GF. Outcome prediction in mild traumatic brain injury: age and clinical variables are stronger predictors than CT abnormalities. J Neurotrauma. (2010) 27:655–68. 10.1089/neu.2009.1059 20035619

[27] de Koning ME Scheenen ME van der Horn HJ Hageman G Roks G Yilmaz T Outpatient follow-up after mild traumatic brain injury: results of the UPFRONT-study. Brain Inj. (2017) 31:1102–8. 10.1080/02699052.2017.1296193 28481634

[28] Lovell MR Collins MW. Neuropsychological assessment of the college football player. J Head Trauma Rehabil. (1998) 13:9–26. 10.1097/00001199-199804000-00004 9575253

[29] Giola G Collins M Acute Concussion Evaluation (ACE): Physician/Clinician Office Version. Centers for Disease Control and Prevention (CDC) (2006).

[30] Lingsma HF Yue JK Maas AIR Steyerberg EW Manley GT TRACK-TBI Investigators. Outcome prediction after mild and complicated mild traumatic brain injury: external validation of existing models and identification of new predictors using the TRACK-TBI pilot study. J Neurotrauma. (2015) 32:83–94. 10.1089/neu.2014.3384 25025611 PMC4291219

[31] van der Naalt J Timmerman ME de Koning ME van der Horn HJ Scheenen ME Jacobs B Early predictors of outcome after mild traumatic brain injury (UPFRONT): an observational cohort study. Lancet Neurol. (2017) 16:532–40. 10.1016/S1474-4422(17)30117-5 28653646

[32] Cnossen MC Winkler EA Yue JK Okonkwo DO Valadka A Steyerberg EW. Development of a prediction model for post-concussive symptoms following mild traumatic brain injury: a TRACK-TBI pilot study. J Neurotrauma. (2017) 10.1089/neu.2016.4819. [Epub ahead of print] 28343409 PMC7647929

[33] Pirracchio R Yue JK Manley GT van der Laan MJ Hubbard AE TRACK-TBI Investigators including Wayne A Gordon. Collaborative targeted maximum likelihood estimation for variable importance measure: illustration for functional outcome prediction in mild traumatic brain injuries. Stat Methods Med Res. (2018) 27:286–97. 10.1177/0962280215627335 27363429 PMC5589499

[34] McMahon P Hricik A Yue JK Puccio AM Inoue T Lingsma HF. Symptomatology and functional outcome in mild traumatic brain injury: results from the prospective TRACK-TBI study. J Neurotrauma. (2014) 31:26–33. 10.1089/neu.2013.2984 23952719 PMC3880097

[35] Gfeller JD Chibnall JT Duckro PN. Postconcussion symptoms and cognitive functioning in posttraumatic headache patients. Headache. (1994) 34:503–7. 10.1111/j.1526-4610.1994.hed3409503.x 8002321

[36] Lew HL Lin P-H Fuh J-L Wang S-J Clark DJ Walker WC. Characteristics and treatment of headache after traumatic brain injury: a focused review. Am J Phys Med Rehabil. (2006) 85:619–27. 10.1097/01.phm.0000223235.09931.c0 16788394

[37] Lucas S Smith BM Temkin N Bell KR Dikmen S Hoffman JM. Comorbidity of headache and depression after mild traumatic brain injury. Headache. (2016) 56:323–30. 10.1111/head.12762 26814846

[38] Solomon S. Posttraumatic headache. Med Clin North Am. (2001) 85:987–96, vii–viii. 10.1016/S0025-7125(05)70355-2 11480269

[39] Lucas S Characterization and management of headache after mild traumatic brain injury. In: Kobeissy FH, editor. Brain Neurotrauma: Molecular, Neuropsychological, and Rehabilitation Aspects. Boca Raton, FL: CRC Press/Taylor & Francis (2015). 10.1201/b18126-16 26269882

[40] Hoffman JM Lucas S Dikmen S Braden CA Brown AW Brunner R. Natural history of headache after traumatic brain injury. J Neurotrauma. (2011) 28:1719–25. 10.1089/neu.2011.1914 21732765 PMC3172878

[41] Esterov D Greenwald BD. Autonomic dysfunction after mild traumatic brain injury. Brain Sci. (2017) 7:100. 10.3390/brainsci7080100 28800081 PMC5575620

[42] Beri N Patrick-Miller LJ Egleston BL Hall MJ Domchek SM Daly MB. Preferences for in-person disclosure: patients declining telephone disclosure characteristics and outcomes in the multicenter Communication Of GENetic Test Results by Telephone study. Clin Genet. (2019) 95:293–301. 10.1111/cge.13474 30417332 PMC6453119

